# Geographic isolation shapes the genetic landscape of the threatened karst-endemic plant *Malania oleifera* (Ximeniaceae)

**DOI:** 10.3389/fpls.2026.1759710

**Published:** 2026-03-04

**Authors:** Ye Zhang, Shuoxing Wei, Zhihui Wang, Feng Gao, Qiujie Lu, Xiaoning Zhang, Qiulan Wei, Dong Lin, Ping Wang, Mimi Li

**Affiliations:** 1 School of Ecology and Environment Science, Central South University of Forestry and Technology, Changsha, China; 2 Guangxi Forestry Research Institute, Nanning, China; 3 Guangxi Laboratory of Forestry, Nanning, China; 4 Guangxi Key Laboratory of Special Non-wood Forests Cultivation and Utilization, Nanning, China; 5 Institute of Botany, Jiangsu Province and Chinese Academy of Sciences, Nanjing, China

**Keywords:** demographic history, genetic diversity, genotyping-by-sequencing (GBS), *Malania oleifera* Chun & S.K. Lee, population differentiation

## Abstract

*Malania oleifera* Chun & S.K. Lee is a rare and endangered tree species endemic to the karst forests of southwestern China. Its seeds are rich in nervonic acid, a compound of significant ecological and economic value. However, habitat fragmentation, overharvesting, and climate change have imposed severe survival pressures on this species, leading to a risk of genetic diversity loss. In this study, we employed genotyping-by-sequencing (GBS) to investigate the genome-wide genetic diversity and population structure of 89 individuals from 16 natural populations. A total of 332,551 high-quality single nucleotide polymorphisms (SNPs) were obtained. The results showed moderate genetic diversity, with populations in Guangxi exhibiting significantly higher nucleotide diversity than those in Yunnan. Population structure analyses identified six genetic clusters that corresponded closely to their geographic distribution, indicating that geographic isolation is the main driver of genetic differentiation. Mantel tests revealed a highly significant positive correlation between genetic and geographic distances but no correlation with environmental distance, representing a typical isolation-by-distance (IBD) pattern. Redundancy analysis (RDA) identified 4,361 SNPs significantly associated with environmental variables suggesting potential local adaptation signals. Demographic reconstruction revealed that *M. oleifera* began a sharp and continuous decline in effective population size approximately 30 kya, likely triggered by climatic fluctuations during the Last Glacial Maximum. These findings provide valuable insights for the conservation, restoration, and regional management of this ecologically and economically important species.

## 1 Introduction

*Malania oleifera* Chun & S.K. Lee, commonly known as the garlic-fruit tree, is a monotypic species of the family Ximeniaceae, endemic to southwestern China. It is an ancient, evergreen, root hemiparasitic tree mainly distributed in the karst mountains along the border of Guangxi and Yunnan Provinces, typically occurring in subtropical evergreen broad-leaved forests at altitudes of 600–1,300 m (Li et al., 2019; Wang et al., 2025). Regarding its reproductive biology, *M. oleifera* exhibits dichogamy and is predominantly outcrossing, depending heavily on insect-mediated pollination (Lai et al., 2008). Its large, oil-rich seeds are dispersed primarily via gravity and scatter-hoarding by rodents (Huang et al., 2008). These restricted reproductive and dispersal mechanisms, compounded by habitat fragmentation, have led to weak natural regeneration and an increasingly shrinking distribution range. Consequently, the species has been listed as a Class II Nationally Key Protected Wild Plant and recognized as a plant species with extremely small populations (PSESP) in China (Ma et al., 2013). It is also categorized as Vulnerable (Vu) on the IUCN (International Union for Conservation of Nature) Red List (IUCN, 1998), highlighting its threatened status and urgent need for conservation. The seeds of *M. oleifera* are rich in nervonic acid (Tang et al., 2013; Wang et al., 2021), one of the highest known concentrations among plant species, conferring high medicinal, economic, and ecological value (He et al., 2022; Li et al., 2025). Thus, it is considered a promising candidate for the discovery and sustainable utilization of nervonic acid resources. However, wild populations face multiple threats, including habitat destruction, overharvesting, and climate change (Zhang et al., 2025), which have exacerbated the risk of genetic diversity loss and inbreeding. Therefore, assessing the genetic diversity and population structure of *M. oleifera* is essential for revealing its adaptive potential, understanding its evolutionary history, and informing conservation strategies.

Geographic environments play a crucial role in shaping species’ genetic structures by influencing dispersal and survival. Rivers, as dominant linear landforms, can act as significant barriers to gene flow, fragmenting habitats and restricting pollen and seed dispersal. Consequently, they reduce cross-river gene exchange and accelerate population differentiation, particularly in subtropical karst mountain regions characterized by rugged terrain and habitat fragmentation (Clements et al., 2006). When gene flow is restricted, populations often exhibit patterns of isolation by distance (IBD) or isolation by environment (IBE) along spatial or ecological gradients (Rousset, 1997; Bohonak, 2002; Sexton et al., 2014; Wang and Bradburd, 2014). Landscape genetics integrates these concepts, combining multi-scale topographic and environmental data to quantitatively assess the relative contributions of gene flow, genetic drift, and natural selection in shaping genetic variation and divergence (Manel et al., 2003; Storfer et al., 2010).

Recent advances in high-throughput sequencing technologies have greatly facilitated molecular studies on *M. oleifera*. Using PacBio long-read and Illumina short-read sequencing, researchers first assembled a draft genome and performed gene annotation, providing a genomic foundation for subsequent studies on nervonic acid biosynthesis and conservation genomics (Xu et al., 2019). Subsequently, by integrating PacBio, Illumina, and Hi-C technologies, a chromosome-level genome assembly of *M. oleifera* was generated, significantly improving assembly quality and enabling the identification of key genes involved in nervonic acid biosynthesis. Whole-genome resequencing analyses revealed that *M. oleifera* maintains relatively high nucleotide diversity compared to other endangered woody plants, but also exhibits high levels of inbreeding and evidence of a historical bottleneck following population expansion, providing valuable insights for extinction risk assessment (Shen et al., 2024).

Compared with whole-genome resequencing, genotyping-by-sequencing (GBS) is a cost-effective, high-throughput reduced-representation sequencing method. GBS employs restriction enzyme digestion and multiplex PCR amplification to target specific genomic regions for single nucleotide polymorphism (SNP) discovery and genotyping, enabling the rapid generation of tens of thousands of molecular markers without requiring a reference genome. This approach is particularly suitable for endangered species with small population sizes, where large-scale, cost-efficient assessments of genetic diversity, population structure, and kinship are needed (Liu et al., 2024; Yadav et al., 2024; Zhang et al., 2024; Lee et al., 2025; Liu et al., 2025). In contrast, while whole-genome sequencing provides comprehensive genetic variation information, it requires greater sequencing depth, computational resources, and cost, making GBS an efficient alternative for conservation genomics applications.

Building on these developments, the present study conducted systematic sampling across the natural distribution range of *M. oleifera* and employed GBS to evaluate population genetic diversity, population differentiation, and gene flow. Furthermore, we examined the relative effects of geographic distance and environmental heterogeneity on genetic structure, identified candidate loci potentially associated with environmental adaptation, and reconstructed historical changes in effective population size. This study aims to provide a scientific basis for the conservation and regional management of *M. oleifera* genetic resources and to compare the performance of reduced-representation (GBS) and whole-genome resequencing approaches in assessing genetic diversity, offering methodological guidance for endangered species lacking reference genomes.

## 2 Materials and methods

### 2.1 Sample collection, DNA extraction, library construction, and sequencing

**Figure 1:**
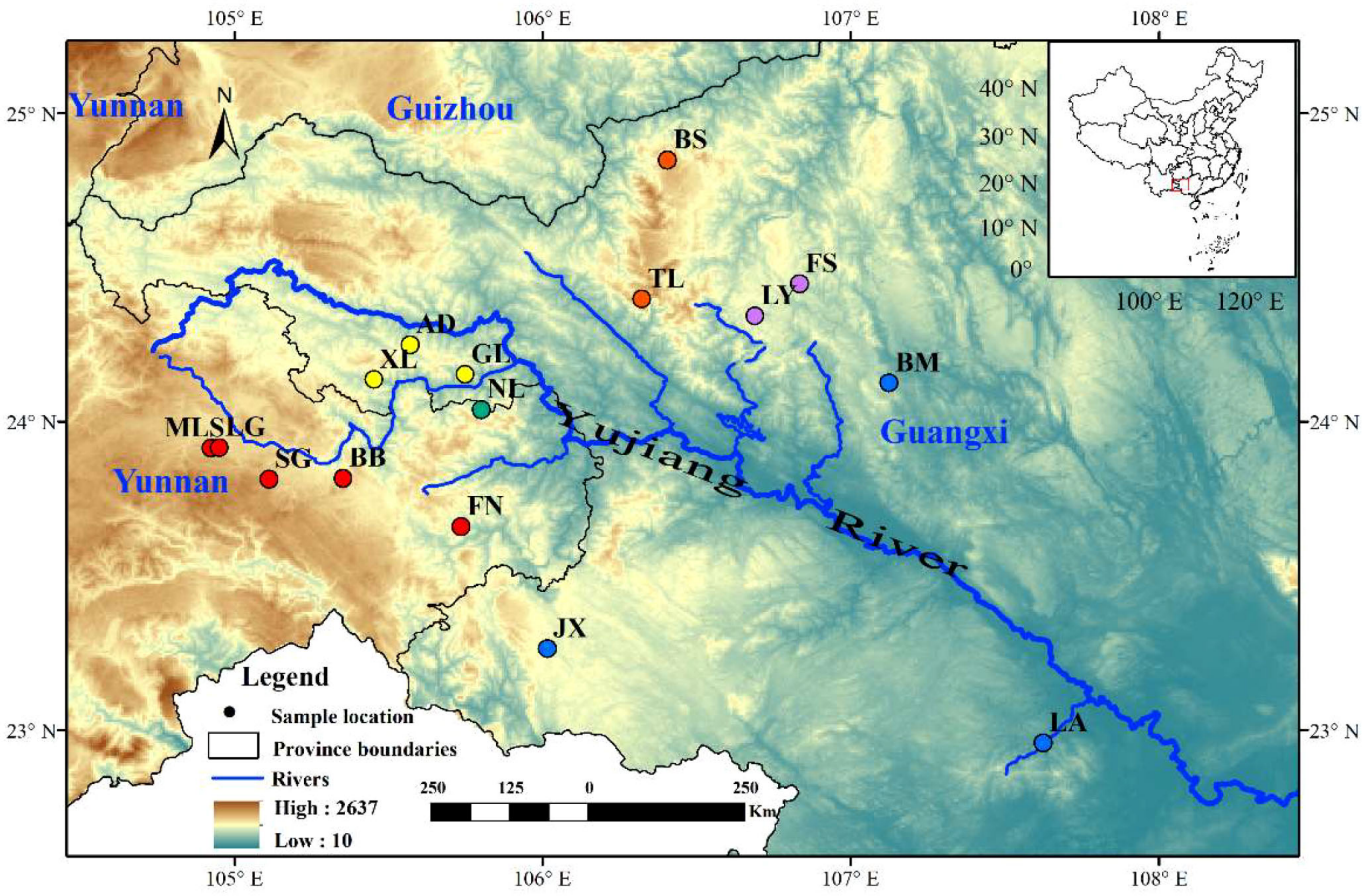
Sampling map of *Malania oleifera*. Colored circles represent distinct genetic clusters identified by population structure analyses. The thick blue line denotes the main channel of the Yujiang River, and thin blue lines indicate its major tributaries.

A total of 89 individuals from 16 populations of *Malania oleifera* were collected, including five populations from Yunnan and eleven from Guangxi (**Figure 1**). Genomic DNA was extracted from silica-dried leaf tissue using a modified CTAB protocol (Doyle and Doyle, 1987). DNA concentration and purity were initially measured using a Nanodrop 1000 spectrophotometer (Nanodrop, MA, USA), followed by accurate quantification using a Qubit fluorometer (Thermo Fisher Scientific, CA, USA). DNA integrity and potential RNA contamination were assessed via 0.8% agarose gel electrophoresis.

High-quality DNA samples were digested with two restriction enzymes, *EcoRI* and *MspI*, and adapters were ligated to both ends of DNA fragments using T4 DNA ligase. The ligated products were amplified by PCR, and 400–600 bp fragments were recovered using 1% agarose gel electrophoresis and purified with AMPure XP magnetic beads. Purified products were sequenced on the Illumina HiSeq 4000 platform (Illumina, CA, USA) with paired-end reads of 150 bp.

### 2.2 SNP calling and filtering

Raw reads were filtered using Fastp (Chen et al., 2018) to remove adapter sequences, abnormal bases, and low-quality reads (reads with ≥10% unidentified bases or ≥50% bases with Phred quality score ≤10). Clean reads were aligned to the *M. oleifera* reference genome (GCA_029873635.1) using BWA (Li and Durbin, 2009) (parameters: mem -t 4 -k 32 -M). The alignment results were sorted and indexed using SAMtools (Weeks and Luecke, 2017), and the average sequencing depth was calculated with the *samtools depth* command. SNP calling was performed using bcftools (Weeks and Luecke, 2017), and the following filtering criteria were applied: minimum sequencing depth ≥3×, missing genotype rate ≤30%, and minor allele frequency (MAF) ≥0.05. The remaining high-quality SNPs were used for downstream population genetic analyses.

### 2.3 Population genetic diversity analysis

Genetic diversity indices, including nucleotide diversity (π), observed heterozygosity (Ho), expected heterozygosity (He), Tajima’s *D*, pairwise genetic differentiation (F_ST_), and gene flow (Nm), were calculated using VCFtools (Danecek et al., 2011).

### 2.4 Population structure analysis

Input files for ADMIXTURE were generated using PLINK (Purcell et al., 2007). Population structure and ancestral components were inferred using ADMIXTURE (Harris and DeGiorgio, 2017) with predefined clusters (K = 1–10). The optimal K value was determined based on the minimum cross-validation (CV) error. The results were visualized using the R package *ggplot2* (https://github.com/tidyverse/ggplot2). Principal component analysis (PCA) was also conducted using PLINK (Purcell et al., 2007), and the results were visualized in three dimensions using the R package *plotly* (https://github.com/plotly/plotly.R). Pairwise genetic distances among individuals were calculated in PLINK (Purcell et al., 2007), and a neighbor-joining (NJ) tree was constructed using the R package *ape* (https://github.com/emmanuelparadis/ape). The NJ tree was visualized and annotated using iTOL (Letunic and Bork, 2024).

### 2.5 Gene flow analysis

Gene flow among populations was inferred using Treemix (Pickrell and Pritchard, 2012). Migration parameters (m) were set from 1 to 10, and each m value was repeated ten times. The optimal number of migration edges was determined using the R package *optM* (Fitak, 2021). Visualization of migration events was performed with the *plotting_funcs.R* script provided in Treemix (Pickrell and Pritchard, 2012).

### 2.6 Isolation by distance and isolation by environment

Pairwise genetic distances were estimated based on F_ST_/(1−F_ST_) using VCFtools (Danecek et al., 2011). To quantify isolation by environment (IBE), environmental distance was calculated using a subset of key variables. Based on the major environmental drivers identified by Zhang et al. (2025), we performed a Variance Inflation Factor (VIF) analysis to minimize multicollinearity. The retained variables (Bio07, Bio15, Bio16, srad, and t_usada_tex) were then used for subsequent analysis. Geographic distance, representing isolation by distance (IBD), was computed from population coordinates using the R package *geosphere*. Partial Mantel tests were performed using the R package *vegan* (https://github.com/vegandevs/vegan) to disentangle the effects of geography and environment. Specifically, IBD was tested while controlling for environmental distance, and IBE was tested while controlling for geographic distance. The significance of correlations was assessed using 999 permutations.

### 2.7 Redundancy analysis

Redundancy analysis (RDA) was conducted using the R package *vegan* to evaluate the influence of environmental variables on genetic variation among SNPs. SNPs significantly associated with environmental factors (p < 0.05) were mapped to the *M. oleifera* reference genome to identify candidate genes.

### 2.8 Demographic history reconstruction

Historical changes in effective population size (Ne) were inferred using Stairway Plot 2 (Liu and Fu, 2020). The site frequency spectrum (SFS) was generated from SNP data using the Python script *easySFS* (https://github.com/isaacovercast/easySFS), with the folded SFS configuration to account for minor allele frequencies. The generation time was set to 10 years, and the neutral mutation rate to 2.5 × 10^-8^ per site per generation (Shen et al., 2024). Projection values maximizing the number of segregating sites were used to output SFS data, which were then provided as input for Stairway Plot analyses. Sixty-seven percent of the sites were randomly selected, and 200 bootstrap replicates were performed to estimate the median Ne and its 95% pseudo-confidence intervals (CIs).

## 3 Results

### 3.1 SNP filtering

**Figure 2:**
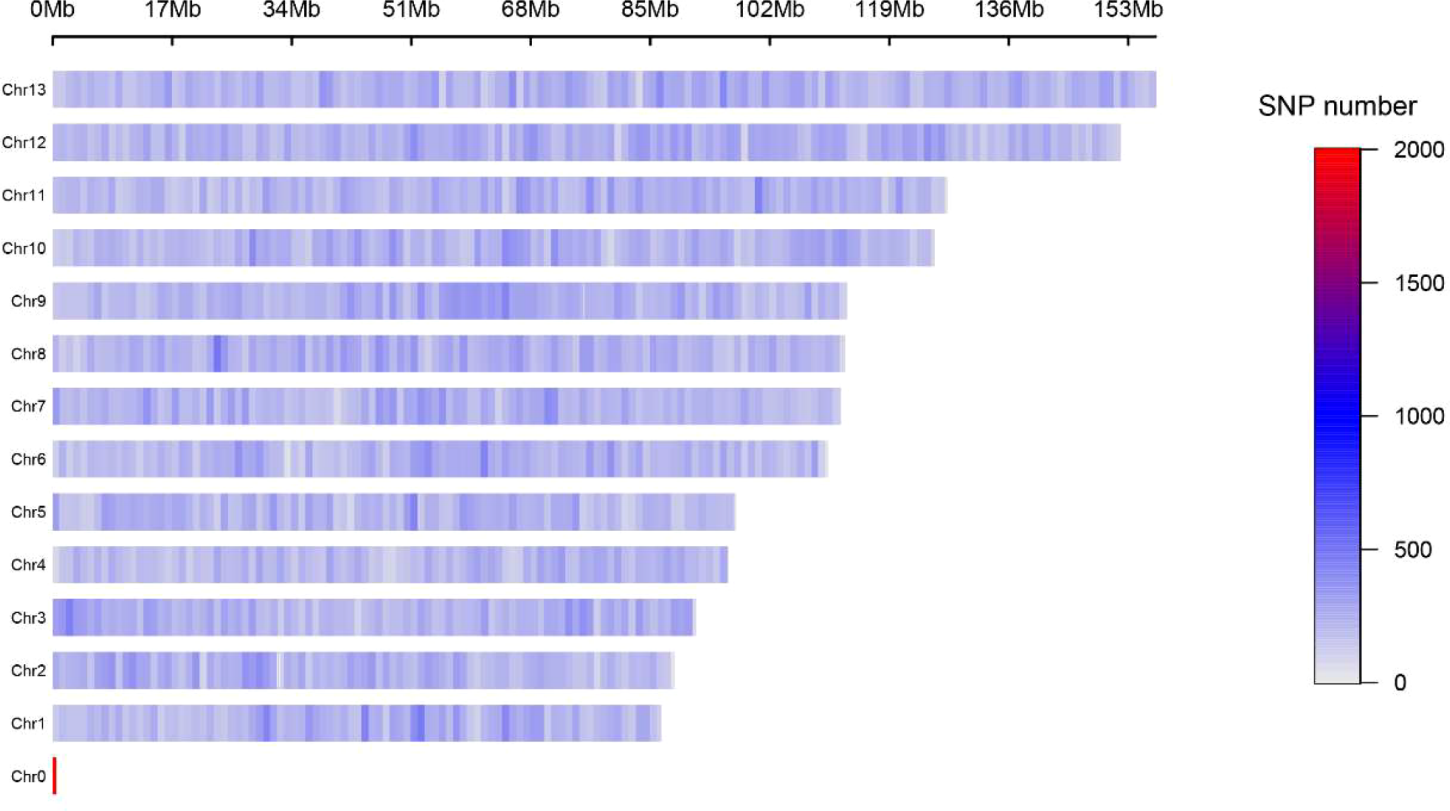
Distribution of SNPs across the reference genome. Chr0 represents unanchored sequences, while Chr1–Chr13 correspond to the 13 chromosomes of *Malania oleifera*.

A total of 16 populations of *Malania oleifera* were genotyped using the GBS approach. In total, 3,278,540 raw SNPs were identified, and after stringent quality filtering and validation, 332,551 high-quality single nucleotide polymorphisms (SNPs) were retained for downstream analyses (**Figure 2**). The raw reads generated in this study have been deposited in the National Center for Biotechnology Information (NCBI) Sequence Read Archive (SRA) under BioProject accession PRJNA1418834 with SRA run accessions SRR37114155-SRR37114243.

### 3.2 Genetic diversity

Genetic diversity indices, including nucleotide diversity (π), observed heterozygosity (Ho), expected heterozygosity (He), and Tajima’s D, were calculated for each of the 16 populations (**Table 1**). At the provincial level, populations from Guangxi exhibited significantly higher nucleotide diversity compared to those from Yunnan. Conversely, no significant differences were observed between the two regions for mean Ho, He, and Tajima’s *D*.

**Table 1:** Collection and genetic diversity information of *Malania oleifera*.

Region	Population	Location	*N*	π	Ho	He	Tajima’s *D*
Guangxi	JX	Jingxi, Baise	1	0.242			
	LA	Longan, Nanning	6	0.225	0.403	0.375	0.619
	BM	Bama, Hechi	6	0.264	0.397	0.373	0.583
	FS	Fengshan, Hechi	6	0.241	0.379	0.350	0.428
	LY	Lingyun, Baise	6	0.227	0.393	0.366	0.531
	BS	Leye, Baise	6	0.197	0.399	0.386	0.584
	TL	Tianlin, Baise	10	0.204	0.351	0.358	0.601
	NL	Nalong, Baise	6	0.184	0.406	0.440	0.670
	GL	Gaolong, Baise	4	0.243	0.431	0.365	0.404
	AD	Anding, Baise	5	0.185	0.418	0.448	0.559
	XL	Xilin, Baise	5	0.132	0.431	0.484	0.654
	Sub-mean		5.5	0.213	0.401	0.395	0.563
Yunnan	FN	Banlun, Funing	6	0.207	0.405	0.378	0.618
	BB	Babao, Guangnan	4	0.186	0.421	0.454	0.317
	MLS	Jiumoli, Guangnan	4	0.109	0.434	0.506	0.446
	LG	Ligan, Guangnan	4	0.136	0.458	0.486	0.572
	SG	Shuguang, Guangnan	10	0.172	0.333	0.304	0.439
	Sub-mean		5.6	0.162^*^	0.410^ns^	0.426^ns^	0.478^ns^

*N*, sample size; π, nucleotide diversity; Ho, observed heterozygosity; He expected heterozygosity; ^*^, p<0.05; ^ns^, not significant.

Nucleotide diversity (π) at the population level ranged from 0.109 in MLS to 0.264 in BM, indicating that the BM population possessed the highest genetic variability, while MLS exhibited the lowest. Observed heterozygosity (Ho) ranged from 0.333 (SG) to 0.458 (LG), whereas expected heterozygosity (He) ranged from 0.304 (SG) to 0.506 (MLS). Most populations showed similar Ho and He values; however, in XL, BB, MLS, and LG, Ho was markedly lower than He, suggesting a deficiency of heterozygotes. This pattern was most pronounced in MLS (Ho = 0.434, He = 0.506) and XL (Ho = 0.431, He = 0.484), implying a potential inbreeding effect within these populations.

Tajima’s *D* values were positive in all 15 populations with sufficient data (ranging from 0.317 in BB to 0.670 in NL), suggesting a deficit of rare alleles and a predominance of intermediate-frequency alleles. Because the JX population included only a single sample (N = 1), Ho, He, and Tajima’s D could not be estimated.

### 3.3 Population structure

**Figure 3:**
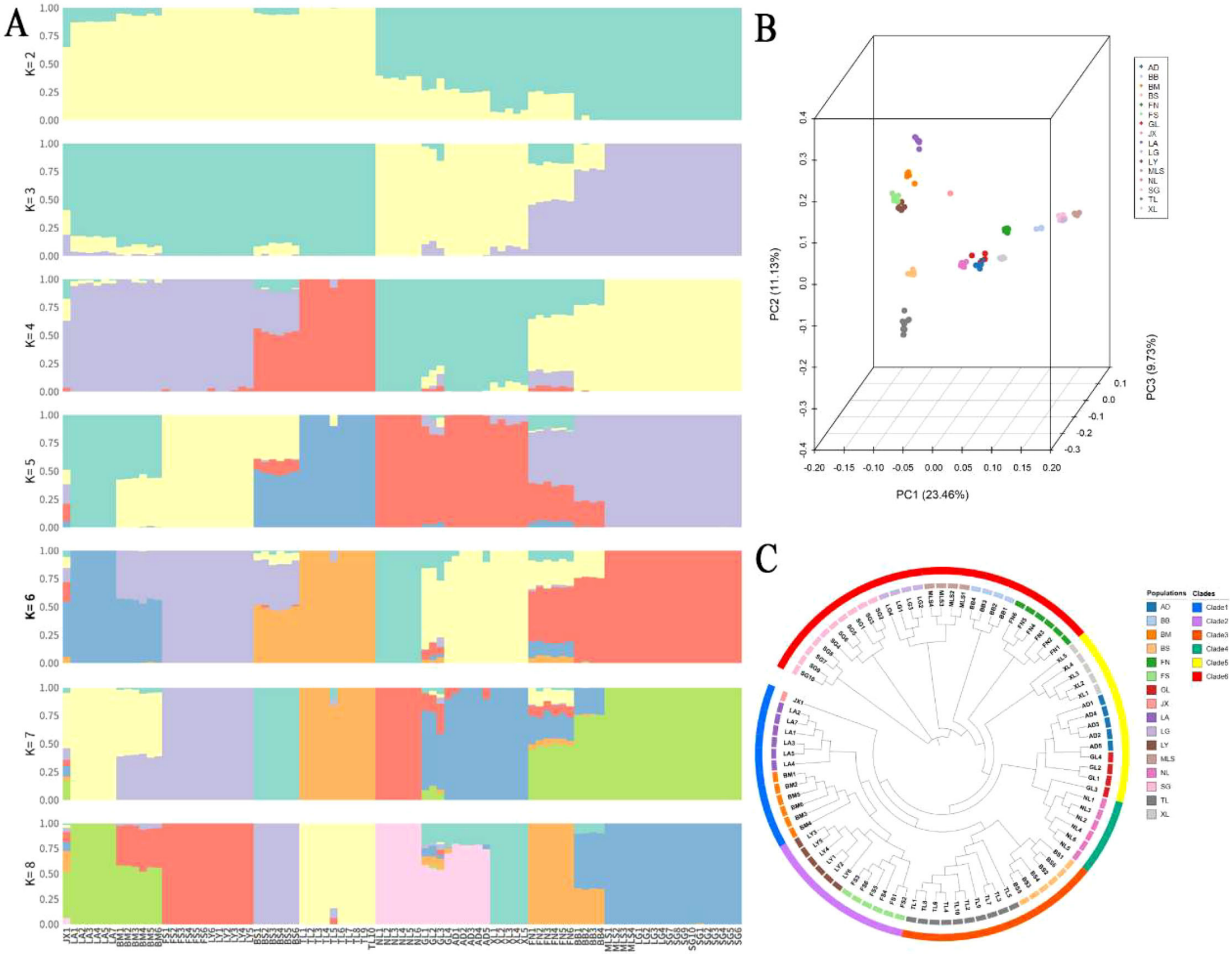
Population structure analysis. **(A)** Admixture results (K = 2 to 8). The x-axis represents sample names, and the y-axis represents the proportion of each subpopulation. The K value indicates the assumed number of subpopulations, and different colors represent different subpopulations. **(B)** Principal component analysis (PCA), where different colors denote different groups. **(C)** Neighbor-joining tree, with colors indicating different groups.

Population structure was assessed using ADMIXTURE, principal component analysis (PCA), and neighbor-joining (NJ) phylogeny based on SNP data. ADMIXTURE cross-validation analysis indicated that K = 6 was the optimal number of clusters with the lowest CV error (**Supplementary Figure 1**). Based on this model, the 16 populations were divided into six major genetic clusters: Clade 1: JX, BM, and LA; Clade 2: LY and FS; Clade 3: BS and TL; Clade 4: NL; Clade 5: GL, AD, and XL; Clade 6: MLS, LG, SG, BB, and FN (**Figure 3A**). PCA results further supported this structure (**Figure 3B**). The first three principal components clearly separated individuals from different geographical regions, consistent with the ADMIXTURE clustering. Similarly, the NJ tree (**Figure 3C**) grouped individuals into six branches, largely corresponding to their geographic origins. Collectively, these analyses indicate that *M. oleifera* exhibits strong geographic structuring and significant population differentiation.

### 3.4 Genetic differentiation and gene flow

**Figure 4:**
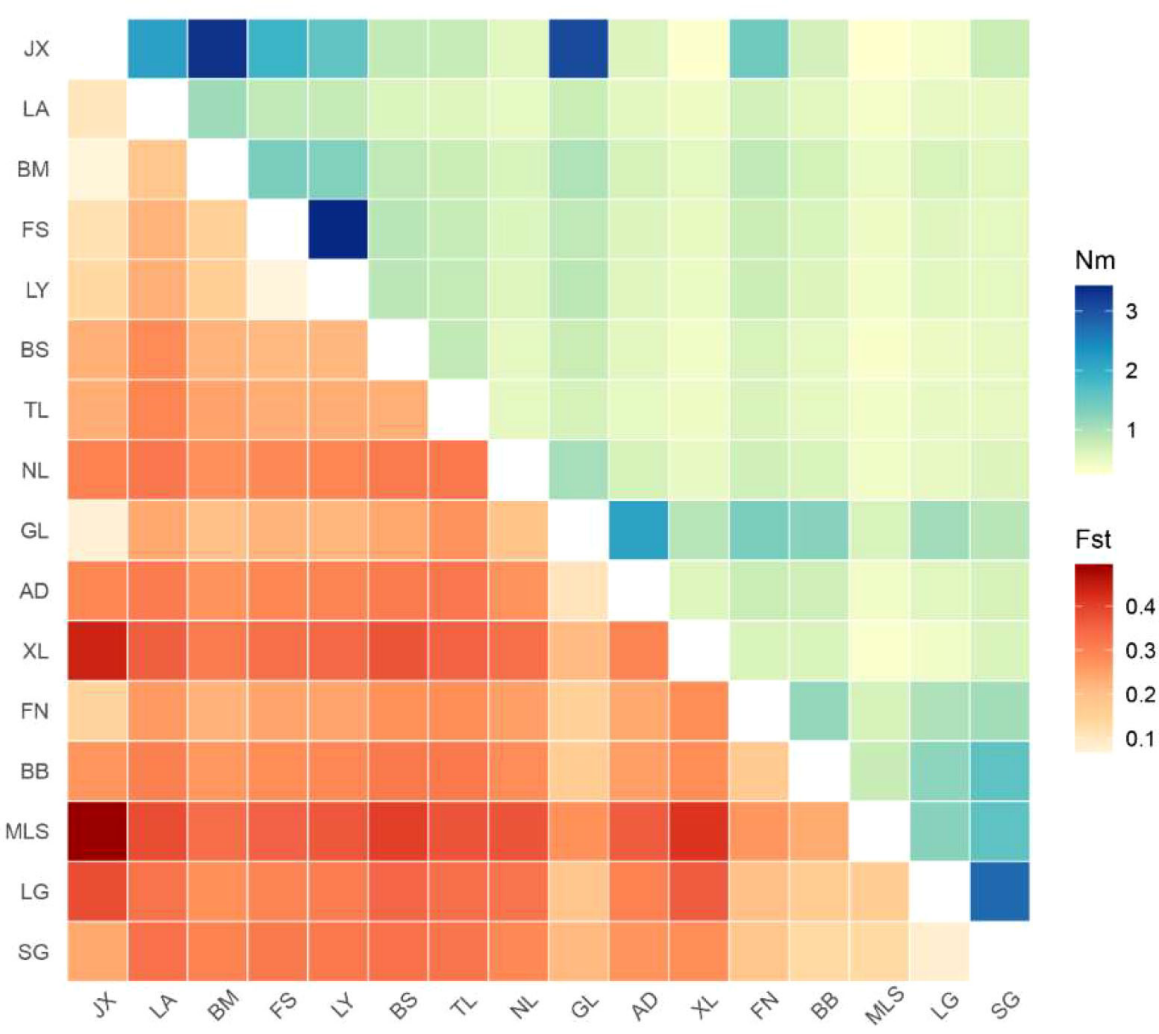
Genetic differentiation (F_ST_) and gene flow (Nm) among populations of *Malania oleifera*. The upper triangle of the matrix represents Nm values, and the lower triangle represents Fst values. The x- and y-axes are labeled with the names of the populations.

Pairwise genetic differentiation (F_ST_) and gene flow (Nm) analyses demonstrated widespread yet variable levels of differentiation among populations (**Figure 4**). F_ST_ values ranged from 0.070 (JX–BM) to 0.495 (JX–MLS). According to Wright’s guidelines (Wright, 1978), values above 0.25 indicate high differentiation; most population pairs exceeded this threshold, reflecting pronounced population structure.

Nm values ranged from 0.255 (JX–MLS) to 3.426 (FS–LY). The majority of population pairs showed Nm < 1.0, suggesting restricted gene flow and a strong effect of genetic drift. The JX–MLS pair exhibited the highest F_ST_ (0.495) and lowest Nm (0.255), indicating strong geographic or reproductive isolation. In contrast, the JX–BM pair displayed the lowest F_ST_ (0.070) and relatively high Nm (3.321), reflecting frequent genetic exchange. Several other pairs also exhibited elevated gene flow (Nm > 2.0), including FS–LY (3.426), JX–GL (3.083), LG–SG (2.762), JX–LA (2.177), and GL–AD (2.131).

**Figure 5:**
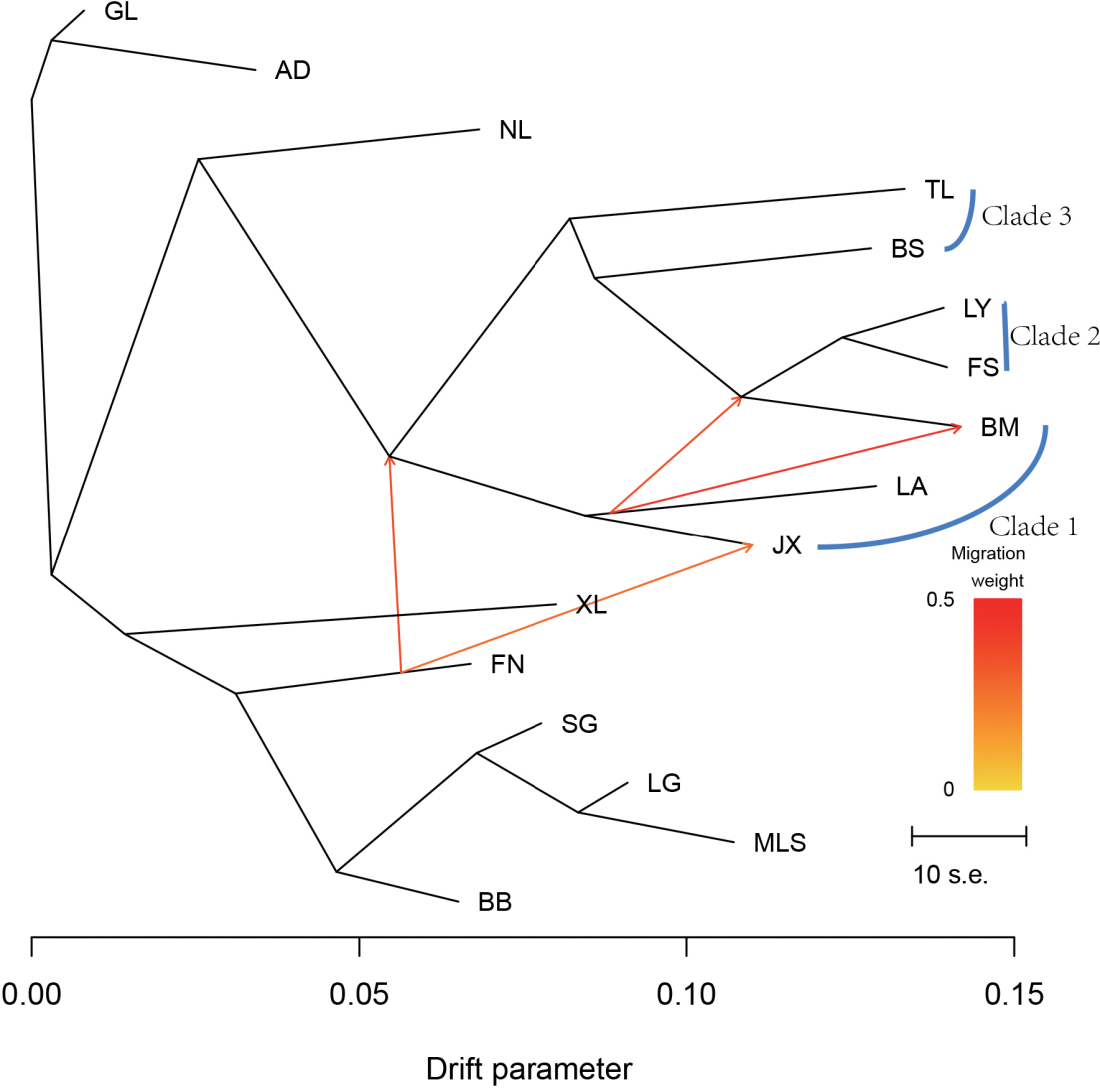
Phylogenetic tree inferred by TreeMix with migration events (m=4). Migration edges are colored according to a heatmap scale to represent migration weights. Horizontal branch lengths represent the drift parameter, indicating the amount of genetic drift.

The optM analysis indicates that m = 4 is the optimal number of migration edges (**Supplementary Figure 2**). Treemix analysis with two migration events (m = 4) revealed a stepwise divergence pattern along the drift axis (**Figure 5**). Four directional migration events were detected: from FN to JX, from FN to Clade 1/Clade 2/Clade 3, from LA to BM, and from LA to LY/FS/BM, mainly among geographically adjacent populations. These findings suggest that localized gene flow still occurs along geographic contact zones despite overall strong differentiation.

### 3.5 Effects of geographic and environmental factors

**Figure 6:**
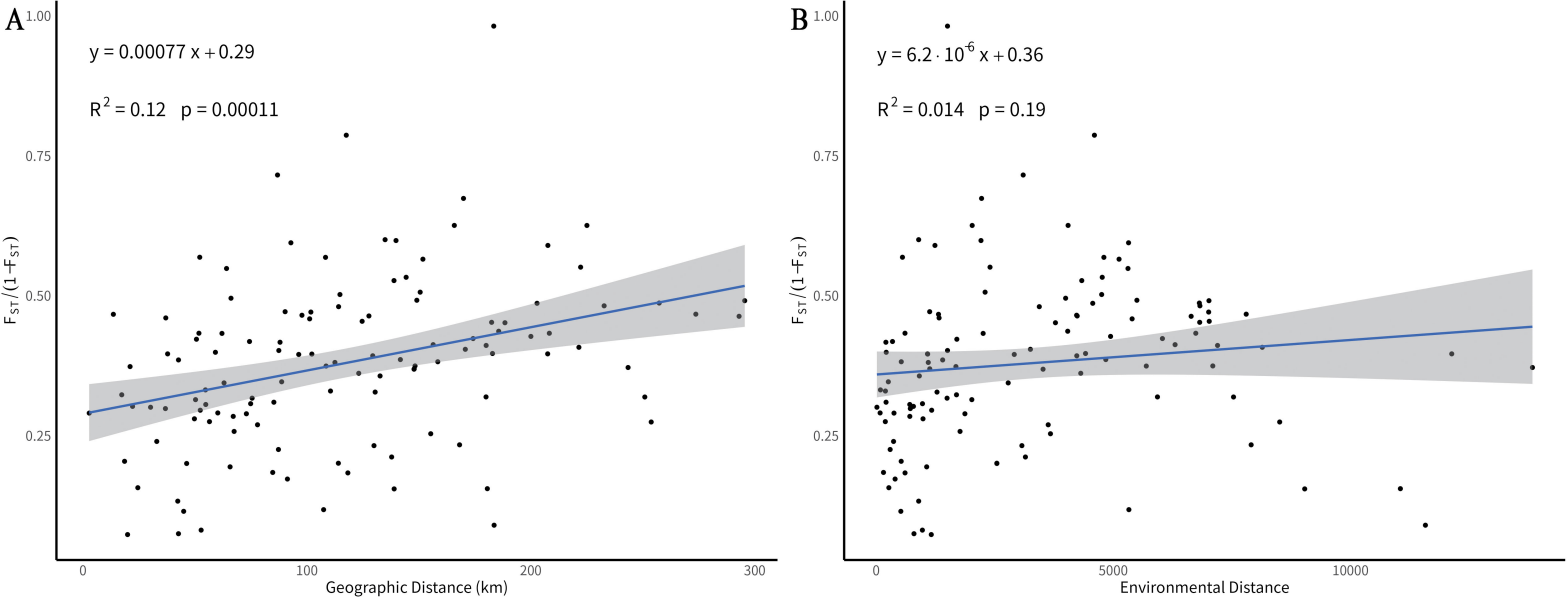
Mantel test of geographic and environmental distances on genetic differentiation. **(A)** Isolation by Distance (IBD). **(B)** Isolation by Environment (IBE). The gray shaded area represents the 95% confidence interval, with the solid line indicating the observed correlation trend.

To identify the main drivers of genetic differentiation, Mantel tests were conducted to evaluate the relationships between genetic differentiation (F_ST_), geographic distance (IBD), and environmental distance (IBE). The results revealed a highly significant positive correlation between genetic and geographic distances (*p* < 0.001; **Figure 6A**), supporting a clear isolation-by-distance (IBD) pattern. This indicates that gene flow is strongly constrained by geographic barriers, leading to greater differentiation among distant populations. In contrast, the correlation between genetic and environmental distances was not significant (*p* = 0.19; **Figure 6B**), suggesting that environmental heterogeneity had a limited role in shaping population structure at this spatial scale. Overall, geographic isolation rather than environmental adaptation appears to be the primary driver of population differentiation in *M. oleifera*.

### 3.6 Redundancy analysis

**Figure 7:**
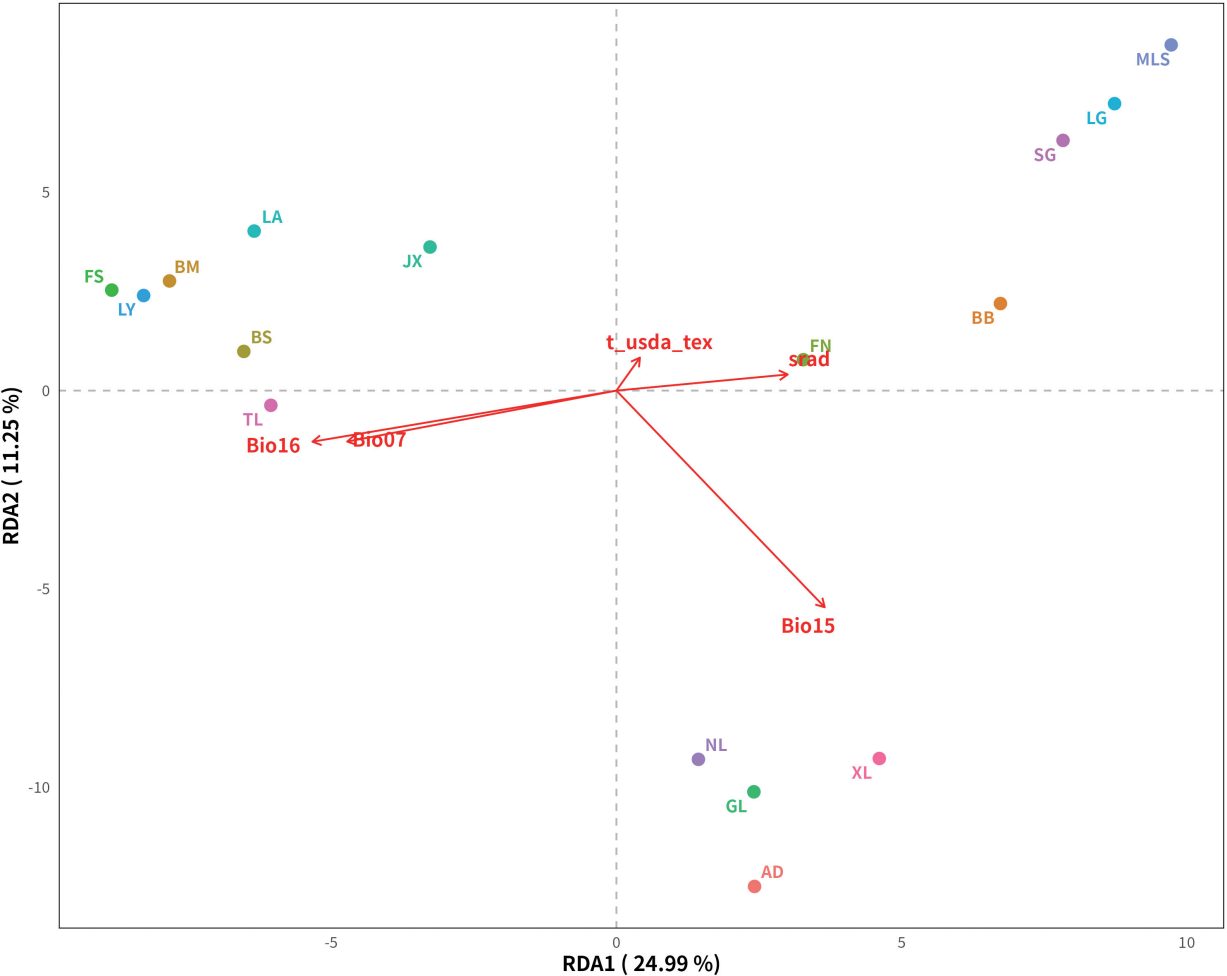
RDA results for *Malania oleifera*. The axes represent constrained axes (RDA axes), points indicate populations, and arrows show the direction and strength of environmental factors. Explained proportions are shown on axis labels.

RDA identified 4,361 SNPs significantly associated with environmental variables, of which 738 (16.9%) were successfully annotated to genes or nearby regulatory regions, while 3,623 (83.1%) were located in noncoding or unannotated regions (**Figure 7**). The main environmental variables included precipitation and soil characteristics. The first two RDA axes cumulatively explained 36.24% of the total genetic variation. These results indicate that precipitation and soil factors may contribute to local adaptation in *M. oleifera*, providing important candidate loci for further Gene Ontology (GO) and Kyoto Encyclopedia of Genes and Genomes (KEGG) analyses and functional validation.

### 3.7 Demographic history

**Figure 8:**
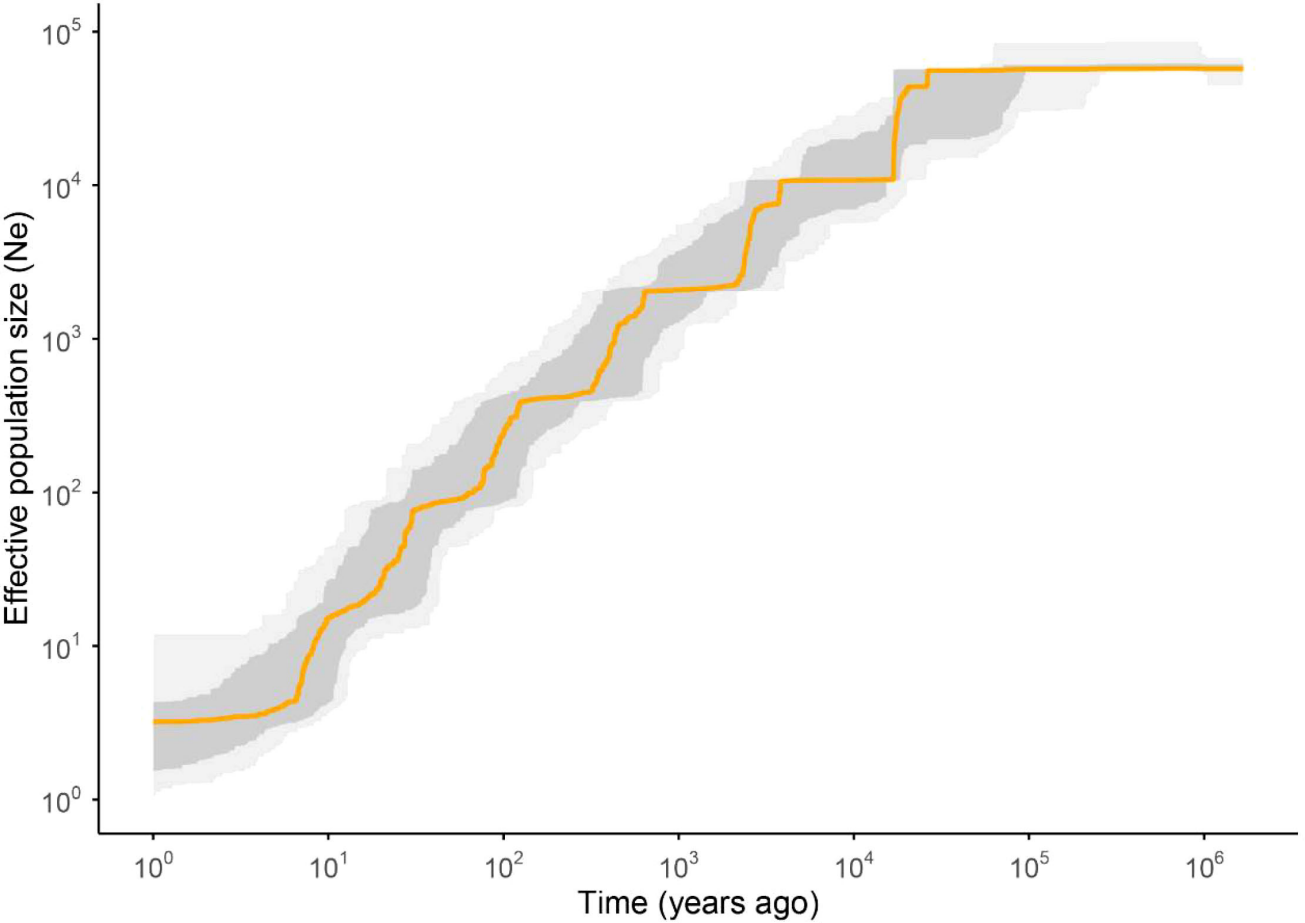
Population historical dynamics of *Malania oleifera*. The x-axis represents the time scale, and the y-axis represents Ne values. The orange line represents the median Ne, and the dark gray and light gray shaded area reflects confidence intervals of 75% and 97.5%, respectively.

Historical demographic reconstruction using Stairway Plot 2 revealed distinct fluctuations in the effective population size (Ne) of *M. oleifera*. In the early period, Ne remained large and relatively stable. Approximately 30 kilo years ago (kya), population size sharply declined, which was likely associated with the onset of and climatic fluctuations during the Last Glacial Maximum (LGM, 21 kya). Thereafter, even with the warming climate during the early Holocene (10–5 kya), the downward trend of the population did not reverse, showing only a slight deceleration in the rate of decline. Subsequently, the population continued to undergo a progressive decline (**Figure 8**).

## 4 Discussion

This study employed genotyping-by-sequencing (GBS) to systematically analyze the genetic diversity, population structure, gene flow, geographic and environmental differentiation, and demographic history of *Malania oleifera* across 16 natural populations in Guangxi and Yunnan. The results revealed a complex genetic pattern shaped within the geographically fragmented karst forests. Overall, *M. oleifera* exhibits a moderate level of genetic diversity, but with pronounced inter-population differentiation and significant spatial genetic structure. This pattern is closely associated with its limited dispersal ability, fragmented habitat, and the influence of historical climatic events.

### 4.1 Genetic diversity and inbreeding

Based on GBS data, *M. oleifera* exhibited a moderate level of nucleotide diversity, whereas genome resequencing revealed a relatively higher level of nucleotide polymorphism (Shen et al., 2024). This discrepancy primarily arises from the methodological differences between the two approaches: GBS captures SNPs only near restriction enzyme cut sites and does not provide uniform coverage across the entire genome, which may lead to an underestimation of genome-wide genetic diversity (Davey et al., 2011; Elshire et al., 2011). Nevertheless, both methods yielded consistent assessments of relative diversity patterns among populations. Regional comparisons indicated that the Yunnan populations exhibited lower genetic diversity than those from Guangxi, while the BM population showed the highest level of genetic diversity, suggesting that it may have retained more ancestral variation or maintained a larger effective population size. In MLS and XL populations, the Ho was lower than He, suggesting heterozygote deficiency and potential inbreeding (Keller, 2002). Such patterns are common in small, geographically isolated plant populations and are usually driven by genetic drift and limited gene flow (Ellstrand and Elam, 1993). As a root hemiparasitic tree species dependent on specific hosts and microhabitats, *M. oleifera* may have intensified inbreeding due to habitat fragmentation and ecological specialization (Li et al., 2019; Wang et al., 2025).

### 4.2 Population structure and geographic differentiation

Population structure analyses consistently revealed *M. oleifera* into six major genetic clusters, which correspond closely with geographic distribution, indicating that geographic isolation plays a dominant role in shaping the genetic structure. The unique geomorphological features of karst regions, including deep valleys, limestone peaks, and narrow gorges, may act as natural barriers that restrict long-distance pollen and seed dispersal, thereby promoting inter-population genetic divergence (Shen et al., 2019). The Yujiang River and its tributary network form a strong dispersal barrier within the karst context, limiting across-river gene flow and accelerating genetic differentiation among river-separated populations (Wu et al., 2024). The river barrier effect has been documented in other mountain and karst species, such as *Parrotia subaequalis* (Hung T. Chang) R.M. Hao & H.T. Wei (Geng et al., 2015), *Primulina tabacum* Hance (Ni et al., 2006) and *Quercus fabri* Hance (Xiong et al., 2020), where inter-river populations exhibit significantly restricted gene flow. Therefore, the genetic structure of *M. oleifera* is shaped not only by mountain isolation but also by the configuration of regional river systems.

High pairwise F_ST_ values indicate strong genetic differentiation, suggesting that gene flow among populations has been largely absent over time. The JX–BM and FS–LY population pairs showed relatively low F_ST_ and higher Nm values, implying localized connectivity. Migration events inferred by Treemix also support the occurrence of historical gene flow among geographically adjacent populations, suggesting that local connectivity may have partially mitigated complete genetic isolation (Perez-Garcia et al., 2025).

### 4.3 Relative effects of geographic and environmental factors

A significant positive correlation between genetic and geographic distances, but no correlation with environmental distance, suggests that the genetic structure of *M. oleifera* is primarily driven by IBD rather than IBE. This pattern aligns with findings in *Fagus crenata* Blume (Hiraoka and Tomaru, 2009) and *Euryodendron excelsum* Hung T. Chang (Shen et al., 2009; Su et al., 2009). *M. oleifera* relies partly on animal-mediated seed dispersal, but due to gravity-dependent seed fall and limited pollination range, gene flow mainly occurs over short distances, resulting in a pronounced spatial genetic gradient (Wu et al., 2004; Huang et al., 2008). Furthermore, geographic barriers such as valleys and rivers intensify isolation effect at the regional scale. Although environmental factors may influence genetic variation locally, they do not appear to dominate the pattern of population differentiation at the spatial scale examined in this study.

### 4.4 Environment-associated loci and local adaptation

RDA analysis identified 4,361 SNPs significantly associated with environmental variables, among which 738 were mapped to known genes or regulatory regions. These candidate loci were primarily related to precipitation patterns and soil properties, suggesting potential signals of local adaptation in *M. oleifera*. Similar environment-driven genetic variation has been reported in other plant species, such as moisture-related adaptive genes in *Cunninghamia lanceolata* (Lamb.) Hook. (Gao et al., 2021) and temperature and precipitation associated loci in *Ulmus elongata* L.K. Fu & C.S. Ding (Lin et al., 2025). Although the overall effect of environmental isolation was not significant, microhabitat variation may still impose selection pressures that shape adaptive differentiation in specific genomic regions, warranting further functional validation.

### 4.5 Demographic history

Demographic inference revealed that *M. oleifera* once maintained a large and stable Ne but experienced a sharp and persistent contraction beginning around 30 kya. This downward trajectory aligns with the climatic fluctuations leading into the LGM (Bai et al., 2018), Unlike many species that exhibited a robust rebound during the postglacial warming of the early Holocene, the population of *M. oleifera* only showed a slowed rate of decline, without a clear recovery. The subsequent and more recent decline in Ne may be further exacerbated by anthropogenic disturbances and increasing habitat fragmentation. This pattern of sustained decline despite climatic amelioration has also been documented in other endangered species, such as *Begonia masoniana* Irmsch. ex Ziesenh. complex (Chen et al., 2024) and *Ostrya rehderiana* Chun (Yang et al., 2018), highlighting the species’ vulnerability to long-term environmental and human-induced pressures.

### 4.6 Conservation implications

From a conservation perspective, *M. oleifera* should be regarded as a geographically isolated species with small and fragmented populations. Conservation strategies, both *in situ* and *ex situ*, should be implemented based on genetic differentiation units, with emphasis on maintaining potential gene flow corridors across rivers and mountain ranges. In addition, conservation and restoration programs should consider local adaptation differences to preserve both overall genetic diversity and adaptive evolutionary potential.

## 5 Conclusion

The genomic landscape of the endangered *Malania oleifera* is a product of long-term geographic isolation and a sustained historical population contraction. Our findings show that the species’ genetic architecture is primarily shaped by its occurrence within fragmented karst topography. In this landscape, physical barriers such as river systems have reinforced an isolation-by-distance (IBD) pattern. This geographic confinement has led to the emergence of six distinct genetic clusters characterized by high differentiation and restricted gene flow. Demographic analysis reveals a sharp population decline that began during the Last Glacial Maximum and persisted into the Holocene without significant recovery. This downward trend was likely worsened by recent human disturbances. While the species maintains moderate genetic diversity, the heterozygote deficiency observed in marginal populations indicates an increased risk of inbreeding and genetic drift. Although environmental selection (IBE) is not the dominant driver of population structure, the identification of adaptive loci associated with precipitation and soil factors suggests that local microhabitats play a vital role in shaping genomic resilience. Conservation strategies should therefore be designed based on geographically and genetically distinct management units, with efforts to enhance potential gene flow among locally connected populations. Furthermore, integrating environmental adaptation information into conservation planning will help maintain genetic diversity and strengthen the adaptive and evolutionary potential of this endangered species.

## Data Availability

The datasets presented in this study can be found in online repositories. The names of the repository/repositories and accession number(s) can be found in the article/**Supplementary Material**.
