# Supplementary material for: Geographic isolation shapes the genetic landscape of the threatened karst-endemic plant Malania oleifera (Ximeniaceae)
Source: Front Plant Sci. 2026 Mar 4;17:1759710. doi: 10.3389/fpls.2026.1759710 (PMC12995749; doi:10.3389/fpls.2026.1759710)
Supplement: Supplementary file 1 [file DataSheet1.docx]

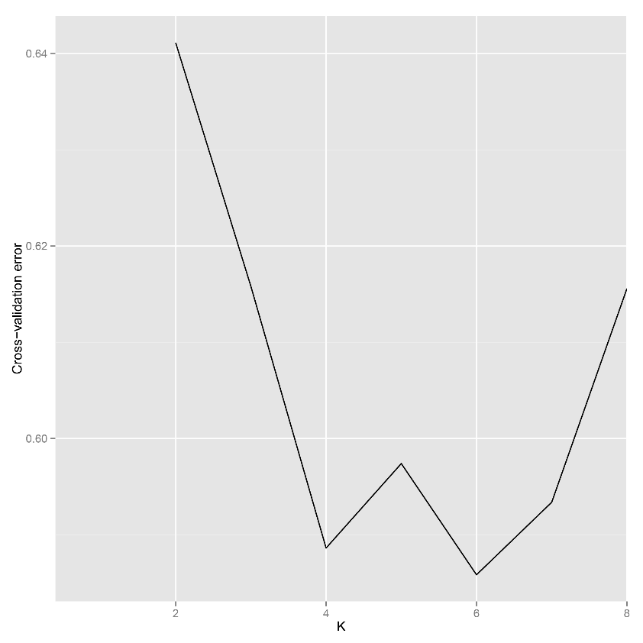


Figure S1. **Cross-validation (CV) error plot for ADMIXTURE analysis.** The plot shows the CV error values for different numbers of potential ancestral clusters (K), ranging from 2 to 8.


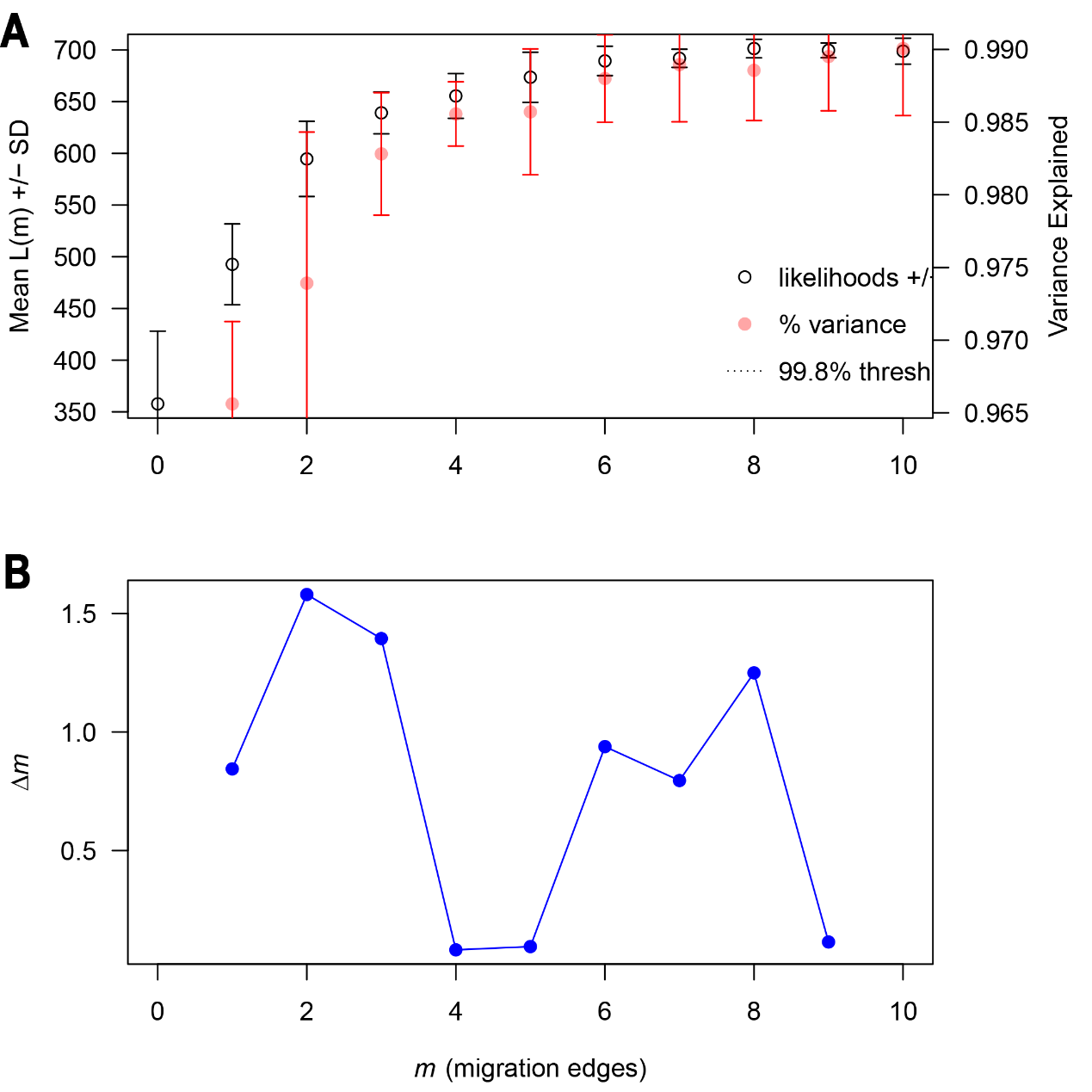


Figure S2. **Model fitting evaluation of migration events in Treemix analysis.** (A) Changes in log-likelihood values (likelihoods) as the number of migration edges (m) increases. Circles represent the mean log-likelihood (Mean L), with vertical bars indicating standard deviation. Pink dots represent the percentage of explained variance (% variance), and the dashed line denotes the 99.8% explained variance threshold (thresh). (B) Changes in the drift parameter (Δm) as the number of migration edges (m) increases.
